# No climate justice without sexual and reproductive health, rights and justice (SRHRJ): past, present, and future challenges faced by the Sexual and Reproductive Health and Rights and Climate Justice Coalition

**DOI:** 10.3389/frph.2025.1569484

**Published:** 2025-08-25

**Authors:** Sally Dijkerman, Heather McMullen, Natalie Hammond, Sexual and Reproductive Health and Rights (SRHR) and Climate Justice Coalition

**Affiliations:** ^1^ Technical Exchange Collective, Ipas, Chapel Hill, NC, United States; ^2^ Global Public Health Unit, Centre for Public Health and Policy, Wolfson Institute of Population Health, Queen Mary University of London, London, United Kingdom; ^3^ Department of Social Care and Social Work, Manchester Metropolitan University, Manchester, United Kingdom

**Keywords:** climate change, sexual and reproductive health and rights (SRHR), reproductive justice, climate justice, coalition, climate action

## Abstract

The climate crisis jeopardizes human health and is one of the greatest threats to reproductive autonomy and human rights. Witnessing these threats, the Sexual and Reproductive Health and Rights and Climate Justice Coalition was formed in 2021 to advocate on the intersections between climate change and sexual and reproductive health, rights, and justice (SRHRJ). The Coalition's purpose is to leverage intersectional approaches to influence global and national policies, programs, and funding mechanisms to advance climate justice, gender equality, and human rights. Climate justice recognizes the climate crisis as a social, political, and environmental problem and demands fair and equitable solutions. Climate action must be participatory, bottom-up, context-specific, and rooted in locally-led evidence and solutions. Achieving climate justice requires achieving SRHRJ. We reflect on three challenges that led to our founding, and which have shaped the work and priorities of our Coalition: understanding and evidencing the links between climate change and SRHR, pushing back on harmful narratives related to population control and the suggestion that limiting reproduction is a climate solution, and finding just solutions to a complex crisis. Comprehensive SRHR empowers communities to be active participants, leaders, and decision-makers in climate action. Therefore, the Coalition continues to advocate for the importance of multisector, collaborative partnerships, oriented around our shared principles of human rights, intersectionality, bodily autonomy, justice, and inclusive organizing, so that SRHR is integrated into all aspects of climate adaptation.

## Introduction

The climate crisis jeopardizes human health and rights at every turn, and sexual and reproductive health, rights, and justice (SRHRJ) are notably under threat. Slow-onset climate changes like rising temperatures and sea levels and rapid-onset climate changes in the form of extreme weather events create and exacerbate intersecting crises that negatively impact sexual and reproductive health and rights (SRHR). This is evidenced in the impacts of climate change such as heat, air pollution and flooding on perinatal health (1–4), the impacts of climate related stress and scarcity on gender-based violence, on disease transmission including HIV (5–8) amongst others. Groups facing multiple and intersecting forms of discrimination are hardest hit demonstrating how climate change exacerbates inequalities in health and rights (6, 9–11). The Guttmacher-*Lancet* Commission on SRHR defines SRHR as a “state of physical, emotional, mental, and social wellbeing in relation to all aspects of sexuality and reproduction, not merely the absence of disease, dysfunction, or infirmity” and “relies on the realisation of sexual and reproductive rights” (12). These rights are based on the human rights to decide whether and when to be sexually active; to decide whether, when, and by what means to have children; and to have one's bodily integrity, privacy, and personal autonomy respected—among others [for the full definition, please see (12)].

SRHRJ, in turn, demands comprehensive “access to a full range of information, services, and support, including comprehensive sexuality education; contraception; menstrual hygiene products and facilities; safe pregnancy; safe birth and support or becoming a young parent; safe abortion; prevention and treatment of HIV/AIDS and other sexually transmitted infections; prevention, detection, and counseling for gender-based violence (GBV); and counseling and care for sexual health and wellbeing” (13). The climate crisis equally undermines the right to have a child, to not have a child, and to parent children in safe and healthy environments, the tenets of reproductive justice (RJ) birthed by racially minoritised feminist women (14, 15). The crisis also violates environmental reproductive justice, in which Mohawk midwife Katsi Cook expanded RJ to include how environmental contaminants threaten physical and cultural reproduction, particularly among indigenous communities (16).

Climate justice (CJ) recognizes the climate crisis as a social, political, and environmental problem. It provides a framework for identifying the true causes and complex impacts of the crisis, while promoting just solutions. First, there are different levels of responsibility for the climate crisis, with those who are most affected being the least responsible. This central tenet of CJ requires the recognition of the climate debt caused by centuries of ongoing colonialism (17) and the disproportionate contributions to greenhouse gas emissions by historically colonizing countries that have captured a majority of the world's wealth (18, 19). Second, climate justice acknowledges that different communities feel the effects of the climate crisis differently, unevenly, and disproportionately. An intersectionality lens is paramount to CJ as it explains that intersecting forms of discrimination and marginalization based on factors that include gender, age, income or social status, ethnicity, disability, sexual orientation, gender identity, gender expression and sex characteristics (SOGIESC), menstruation, and migration, act to create and heighten vulnerability to climate impacts and undermine the ability to cope and adapt. Third, CJ calls for the redress of these injustices in fair and equitable ways, with solutions that lessen impacts on marginalized communities through both emissions reductions and power redistribution in society (20).

Witnessing the myriad impacts of the climate crisis on human rights and SRHR inspired the formation of the SRHR and Climate Justice Coalition in 2021. Co-convened by four organizations—Ipas, Women Deliver, Women's Environment & Development Organization (WEDO), and the Asian-Pacific Resource & Research Centre for Women (ARROW)—we are a global network of more than 100 civil society organizations across over 50 countries committed to collective action and advocacy to advance SRHR and gender equality in the context of climate change. The purpose of the Coalition is to leverage intersectional and climate justice approaches to influence policies, programs, and funding mechanisms that advance climate justice, gender equality, and human rights. Members of our coalition include representatives of women's rights, feminist, SRHR and CJ organizations, movements, advocacy groups, and research and academic institutions.

The climate crisis is a great threat to reproductive autonomy and human rights. Therefore, no discussion of SRHR nor reproductive justice is complete without recognizing the impacts of the climate crisis and related injustices. Achieving climate justice requires achieving SRHRJ, and vice versa. We reflect on three key challenges that led to the founding of the Coalition and which have shaped our work and priorities. These are: understanding the links between climate change and SRHR; resisting harmful population narratives; and finding just solutions to a complex crisis.

### Enough evidence to act: understanding the links between climate change and SRHR

Around the time of our Coalition's forming in 2021, the dearth of published scientific literature exploring connections between climate change and SRHR was a major barrier to fulfilling our purpose. Yet, we were bearing witness to these impacts firsthand in our communities and work across countries and disciplines, especially in the Global South. A primary objective of the Coalition has been to document and communicate the impact of climate change on SRHR, including the identification of gaps in research, programs, funding, and service delivery.

Direct impacts on SRHR are seen from extreme heat, air pollution, increased salinity of water sources, and extreme weather events, all symptoms of climates in crisis. Heat and air pollution directly worsen maternal and neonatal health outcomes, including but not limited to prematurity, low birthweight, stillbirth and neonatal stress (2, 21–25). Increased salinity of freshwater sources in coastal areas due to rising sea levels has been linked to increased hypertension and preeclampsia, with a concomitant increase in miscarriage (26, 27).

The climate crisis also impacts the social and environmental determinants of health, including but not limited to economic livelihoods, food and energy systems, access to clean water, and education, thereby undermining individuals’ access to essential information and health services. These impact pathways are complex, both acute and chronic. For example, climate change is increasing the frequency and intensity of extreme weather events (28), such as cyclones, hurricanes, and wildfires, and the impacts of these events hit those with multiple and intersecting vulnerabilities hardest. Women are more vulnerable to sexual and gender-based violence (SGBV) in storm shelters and often lack the agency and ability to access medical care, including SRH and support services for victims of SGBV (29–32). While understudied, this likely applies to gender-diverse people as well. Further, disasters can interrupt access to contraception, people may forget pack contraceptives when evacuating, contraceptives may be washed away or destroyed by flooding, and people may be unable access contraception due to closed or inaccessible health centers and pharmacies. These factors increase unplanned pregnancies while simultaneously decreasing access to options like emergency contraception, safe abortion and prenatal care (30, 31, 33).

The climate crisis is undermining decades of progress in sustainable development and global health (34). For menstruating people, water scarcity and contamination can reduce the ability to access and maintain menstrual hygiene (30, 31, 35), contributing to gynecological infections. Furthermore, climate change can affect the age of first menses by affecting food availability and via increased air toxin and pollutant exposures (36). Worsening economic and food insecurity resulting from climate change exacerbates existing inequalities for many social groups; for example, by interrupting educational opportunities for girls and exacerbating the drivers of SGBV, reproductive coercion, child, early and forced marriage and unions (CEFMU), and poor mental health (6, 37–41).

These and numerous other impacts of the climate crisis are contributing to rising climate and eco-anxiety globally, defined as distress concerning the climate crisis (42, 43). A survey of 10,000 children and young people across ten countries found disturbingly high levels of climate anxiety impacting daily functioning and threatening mental health (42). For instance, 39.1% of those surveyed reported hesitance to have children due to eco-anxiety (42). People of all ages and sociodemographic backgrounds have expressed desire to control their fertility to help them cope and adapt to the climate crisis (31, 44–46). Qualitative research in coastal Bangladesh communities heavily impacted by climate change-induced cyclones found diverse impacts on women's fertility preferences, with some wanting to delay childbirth following extreme weather events and others desiring more children to protect from childlessness (30). At a time when the demands on individuals to adapt and remain resilient are rapidly increasing, so too are the threats to reproductive and bodily autonomy.

Not all findings are dire, however: emerging evidence also points to clear benefits of realizing comprehensive SRHR as a basis for climate action (47). Investments in SRHR can lessen the impacts of climate disasters and stressors on communities and improve individuals’ adaptive and resilience capacities. For example, building the resiliency of health systems that promote SRHR using frameworks like the World Health Organization's *Framework on integrated, people-centered health services* (48) can reduce the impacts of climate disasters and stressors and build preparedness by bolstering disaster response systems. Interventions like comprehensive sexuality education (CSE) and implementation of the Minimal Initial Service Package (MISP) for sexual and reproductive health in crisis contexts can further reduce negative impacts of the climate crisis on SRHR (47, 49, 50). Case studies from Tanzania, sub-Saharan Africa, and Bangladesh have linked SRHR interventions to improved gender equality and climate resilience (47, 51–54).

An appreciation of these complex interlinkages leads to a simple conclusion: while the evidence base merits further advancement, we have the scholarly evidence to conclude what feminist climate justice advocates had long recognized: the climate crisis is a reproductive justice and human rights crisis. We know that SRHR is fundamental to building healthy communities and advancing gender equality. Both are important facets of building climate resilience. To better act on this knowledge, the SRHR and Climate Justice Coalition emerged, recognizing the need for a space where we could break down silos between the SRHR and climate justice organizations and movements, facilitate knowledge sharing, jointly mobilize, and amplify the voices and priorities of grassroots organizations.

### Resisting harmful population narratives: adopting human rights and reproductive justice approaches

A key challenge for the Coalition has been pushing back on harmful narratives related to population control and the suggestion that limiting reproduction is a solution to the climate crisis. Overpopulation has been a longstanding site of blame for social and environmental ills, which has been reinvigorated in the face of climate change (55). The assertion that climate change can be mitigated by controlling fertility has been continually critiqued, largely by reproductive justice scholars (55–58). This criticism is partly because of the unfair burden this framing places on the people and areas most affected and least responsible for the climate crisis. Climate-related narratives about population also tend to focus on demographic aims and racist population narratives which position people from the most climate affected areas, primarily women, and their bodies as sites of intervention.

Populations in the Global South should not be made to feel responsible for mitigating climate change through reducing their fertility. This suggestion revives and reinvigorates longstanding narratives that suggest the reproduction of some groups of people is problematic and undesirable (55, 56, 58). It also ignores the fact that per capita greenhouse gas emissions in high income countries—the root cause of the crisis—are more than 30 times that of low income countries (19), and these same countries have the lowest fertility rates globally (18). Coercive population policies have been the source of numerous historic and ongoing human rights abuses (70). The reproductive justice movement, which calls for a greater acknowledgement of the structural drivers of inequality, emerged in part in response to racist historic and ongoing policies that suppressed and threatened bodily autonomy (14). Population narratives allocate blame and place the burden of intervention in the Global South and onto the bodies of people who are already suffering the most severe consequences of the colonialism, imperialism, extractivism, militarism, racism, and patriarchy that are driving our current climate catastrophe, rather than addressing unjust systems of consumption and oppression.

Family planning, which the WHO describes as allowing people to attain their desired number of children, if any, and to determine the spacing of their pregnancies, is not a solution to climate change, and approaches that suggest it is are harmful and distracting from the true drivers of the crisis. The Coalition has been previously invited to partner with organizations who profess similar values but suggest the promotion of family planning as a strategy to mitigate emissions, forgoing a wider understanding of SRHR and core principles of RJ to focus only on fertility. Even when couched as “voluntary” family planning or described as rights-based, a family planning approach that ultimately prioritizes the instrumental goal of reducing population growth undermines bodily autonomy and reproductive justice. True climate justice and rights-based approaches, including enhancing the resilience of individuals and communities to the climate chaos destroying their landscapes and livelihoods, rely on the realization of full SRHR. Essential SRHR extends far beyond family planning, as defined in the introduction (12).

Coalition members have developed a messaging guide as a tool to support a rights and justice-based framing of the intersections [see (59)], as well as position statements. This tool is especially helpful for those who might be reticent to engage with the climate-SRHR nexus, and for those who are interfacing with them. Members have also delivered training and clarified what we feel to be the best articulation of the intersections for engendering more justice and meaningful inclusion in climate policy. Controlling bodies will not solve the climate crisis. Climate justice requires being accurate, articulate, and unabating about the true drivers of the crisis, and holding the right actors to account. We have no hope of developing a more just and resilient society out of this time of collapse without upholding SRHR and working to ensure that even the most oppressed and stigmatized in our society can realize their rights. Bodily autonomy is climate resilience, and addressing intersectional inequalities and injustices is essential for achieving climate justice.

### Just solutions to a complex crisis: challenging oversimplification and maladaptation with context-specific, locally-led evidence and solutions

Despite significant progress, a growing challenge for the Coalition and like-minded advocates is the lack of known solutions for successfully integrating SRHR and climate action that supports and advances reproductive justice. This is in part due to the relative infancy of the field (intersection of SRHR and CJ), a lack of rigorously evaluated programs, and the challenges of sharing information and solutions. The linkages between SRHR and climate change are complex and vary widely across settings and populations, yet traditional development and global health strategies tend to gloss over these nuances and value scalable solutions using top-down approaches. Building rigorous evidence for integrated solutions is challenging in part due to the complexity and diversity of climate adaptation and resilience monitoring, learning and evaluation approaches in general; for examples, see (60–63). There are also many practical obstacles to integrating SRHRJ and climate action initiatives at the implementation level. Political and sociocultural realities require some SRH efforts to adopt public health-based rather than rights-based or justice-rooted approaches. Furthermore, limited resources and donor investment manifest in competition among climate and SRHR actors, hindering integrated approaches.

Traditional approaches without an appreciation of intersectionality or integration across disciplines risk maladaptation, whereby actions intended to reduce the impacts of the climate crisis conversely create more risk and vulnerability. Perhaps most notably, climate mitigation strategies that center on limiting reproduction to decrease greenhouse gas emissions violate human rights, as discussed previously. We have therefore found it necessary to challenge the oversimplifications and ignorance of climate impacts on SRHR and advocate for an intersectional perspective in both evidence generation and climate action. We know that factors that create barriers to the realization of RJ also exacerbate vulnerability to climate change and vice-versa. Beyond gender, factors such as sexual orientation, race, indigeneity, ethnicity, age, citizenship status, socioeconomic status, and disability may also be significant (47). The lens of intersectionality demands that we understand and embrace these complexities.

A promising approach to identifying just solutions is Nepal's Local Adaptation Plans of Action (LAPA) Framework for advancing climate adaptation. Such frameworks, while not without critique [see (64)], are a key mechanism for localizing Nepal's National Adaptation Plan (NAP) at the community level, whereby the process is designed to be participatory and community-driven. Local stakeholders, including women, young people, and marginalized groups, are involved in identifying climate risks and vulnerabilities and developing adaptation measures. This structured approach, which embeds core principles of bottom-up planning, inclusivity, responsiveness and flexibility, aims to identify local needs and direct resources to where, when and by whom they are most needed. Additional solutions-oriented case studies from Kenya, the Philippines, India, and beyond have been documented by Coalition members to inspire investment and innovation in this area [please see (65)]. Since 2015, the Women and Gender Constituency has collated “Gender Just Climate Solutions”; examples include MISP provision by the All India Women's Conference and SRH service provision alongside conservation work with Mayan and Q'echqi adolescent girls in Guatemala (66). Coalition members also share their locally-led work during member meetings; the work of documenting and cataloguing the diversity of approaches, activities, and organizations continues to take place in civil society spaces and grey literature.

To achieve progress despite imperfect evidence, Coalition members have adopted approaches to program design, management, and evaluation that center intersectionality. Specifically, feminist participatory action research, Monitoring, Evaluation, Research, Learning, and Adapting (MERLA), and program management are central. Such approaches examine why and how inequalities exist and are reproduced. Core principles include shifting power, being gender transformative, intersectionality, maintaining strict ethical standards, and reframing the role of evaluator or researcher. By centering communities as collaborators and leaders in the creation of knowledge, not just passive recipients, feminist MERLA aims to dismantle the power dynamics underpinning social and political structures that systematically disadvantage women in all their diversity (67, 68).

We need systems approaches that break down silos between sectors, incorporating insights from a wide range of disciplines, and which value processes equally to outcomes—specifically, recognizing that inclusivity, participation, and equity are vital. In summary, for climate action to adequately and successfully uphold and advance SRHR and RJ, efforts must be participatory, bottom-up, context-specific, and rooted in locally-led evidence and solutions.

## Conclusion

As we reflect on these challenges and lessons learned, the SRHR & CJ Coalition stands firm in our knowledge that climate justice cannot be achieved without SRHRJ. Our call to action revolves around, first, the need for greater awareness and stronger collaboration, and second, strengthened, integrated policies. We urge SRHR and climate adaptation practitioners to build awareness of this important intersection across their respective fields. A practical step towards this end is starting within their own organizations and institutions, by reflecting on how the climate crisis is impacting SRHRJ in the communities where they work. We recommend this reflection be done in collaboration with communities, using feminist MERLA and/or participatory action research methods. Rather than SRHR practitioners becoming experts in climate adaptation or vice versa, we call for the development of more partnerships and collaborations across sectors and institutions aimed at finding system-level solutions using pooled resources, so that each sector can benefit from each other's expertise. Fundamentally, we push for both fields to center those most impacted in identifying impacts and solutions, ensuring local leadership, and adopting the principles of climate justice.

Equally important is to advocate for strengthened, integrated policies. Specifically, SRHR actors can support ambitious climate action in countries’ Nationally Determined Contributions to limit global warming below 1.5°C. They can also advocate and partner with health systems to strengthen the capacity of the health workforce on climate change and disaster preparedness, and related policies that support this goal. Climate and SRHR actors alike can support advocacy for integration of SRHR in countries’ National Adaptation Plans (NAPs) and Health NAPs. For additional policy recommendations, please see the Coalition's full call to action (69).

Full realization of SRHR using human rights and reproductive justice approaches is essential to climate resilience, as bodily autonomy enables individuals and communities to adapt to climate change. Comprehensive SRHR supports communities to be active participants, leaders, and decision-makers in climate action. Multisector, collaborative partnerships, oriented around our shared principles of human rights, intersectionality, bodily autonomy, justice, and inclusive organizing, are integral for climate adaptation. We encourage SRHR, climate justice, and reproductive justice organizations to join us in this movement, so that SRHRJ can be made central to climate action efforts globally.

## Data Availability

The original contributions presented in the study are included in the article/Supplementary Material, further inquiries can be directed to the corresponding author.
